# A first psychotic episode with kinesthetic hallucinations. Report of a case

**DOI:** 10.1192/j.eurpsy.2021.2101

**Published:** 2021-08-13

**Authors:** F. Cartas Moreno, M. ValverDe Barea

**Affiliations:** 1 Hospital De Úbeda, Unit Mental Health, Ubeda, Spain; 2 Unit Mental Health, Complejo Hospitalario Jaén, Jaén, Spain

**Keywords:** psychosis, PSYCHOPATOLOGY, delusions, KINESTHETIC

## Abstract

**Introduction:**

It reveals a case that occurred in a patient with no previous history whose first manifestation was kinesthetic allucinations, subsequently appearing other psychopathological alterations

**Objectives:**

24-year-old male. Truck driver by profession. No prior mental health story. Good operation prior to the consultation. He comes accompanied by his parents for having a sensation of having bugs under his skin. It has come to throw gasoline on top to eliminate the possible infection.

**Methods:**

Exploration: He is concius, oriented, with scratching lesions. He wears a cap soaked in gasoline to ward off critters. He does not present in the foreground other psychopathological alterations. CT with normal results, thyroid hormones, and normal biochemistry are requested. Treatment with aripiprazole is initiated in ascending doses, as it presents in the beginning a torpid evolution with the appearance of delusions of injury to its boss.

**Results:**

After that, he is currently psychopathologically stable and has returned to work with his father in the field.

**Conclusions:**

Although not the most common, psychotic disorders can occur at the beginning with cenesthetic alterations. Organic screening should be performed and results treated accordingly.

**Disclosure:**

No significant relationships.

